# Biology Open: evaluating impact

**DOI:** 10.1242/bio.016147

**Published:** 2015-12-16

**Authors:** Rachel Hackett, O. Claire Moulton, Jordan W. Raff

**Affiliations:** 1 The Company of Biologists, Bidder Building, Station Road, Cambridge CB24 9LF, UK; 2 Sir William Dunn School of Pathology, University of Oxford, South Parks Road, Oxford OX1 3RE, UK

## Biology Open: the story so far

BiO is published by The Company of Biologists – a long-established not-for-profit organisation with publishing at its heart. Our four sister journals – important community publications – should be well known to you: Development, Journal of Cell Science, Journal of Experimental Biology and Disease Models & Mechanisms (http://www.biologists.com/journals.html). The Company Directors (http://www.biologists.com/about-us/), all research-active biologists, together made the decision to launch BiO in 2011. They identified the need for a journal that strives to support the members and researchers of the biological sciences community by publishing sound research without a requirement for novelty or impact (Hunt and Moulton, 2012). Editor-in-Chief Jordan Raff perceived BiO as part of the solution to a system that is “no longer fit for purpose” (Raff, 2012).

**Fig. 1.**
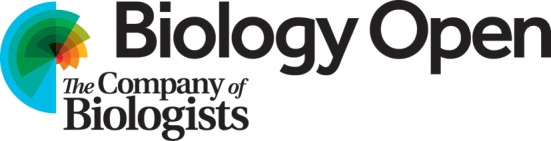
**BiO and The Company of Biologists: Supporting biologists, inspiring biology.**

Since its launch, BiO has published more than 600 good-quality papers that have been accepted on the basis that they are technically sound and their conclusions are supported by the data shown, rather than on the perceived importance of the findings. Regular readers of BiO will have noticed a series of gradual changes during 2015, culminating in the implementation of a new brand for The Company of Biologists and its journals (Fig. 1), and the redesign of the journal website. These new websites are the result of a massive project to ensure that users have an enhanced experience when visiting our pages. The new BiO website is quick, uncluttered, and easy to search and find the content you need. We hope it looks good too.

Although our five journals are well known, fewer people are aware of the other areas of support that The Company of Biologists brings to the biological community. Our new brand will help us to increase the awareness of our work, and strengthen the links between our journals and charitable activities.

## Charitable activities of The Company of Biologists

As a charity, The Company of Biologists uses the surplus it generates for the benefit of biology and the biological community. The Company has made substantial contributions to the community, using funds to organise and facilitate scientific meetings, build and develop communities of biologists, assist the activities of specialist societies and give financial support to young researchers. This investment is overseen by the Board of Directors, who give their time to The Company without payment. They are experienced senior scientists from a range of life science and clinical research backgrounds, who believe in the importance of what The Company does and who are dedicated to furthering its influence. Visit our web pages to find out how to apply for support for workshops and conferences (http://www.biologists.com/grants/). Organisers may use a grant from The Company in a variety of ways, for example, to meet the expenses of a plenary or keynote speaker, assist with travel for early career scientists, or to reduce (or waive) registration fees.

The Company of Biologists also hosts and funds a series of workshops, which are carefully organised to provide leading experts and early career scientists from a diverse range of scientific backgrounds with an inspiring environment for the cross-fertilization of interdisciplinary ideas. We are always looking for new and exciting topics for workshops; visit http://www.biologists.com/workshops/propose-new-workshop/ to find out more and read our archive of information about previous workshops.

## The world of Open Access publishing

Authors rightly have expectations of a journal. And, for an Open Access journal, whose authors are the principal customer (as opposed to subscribers), perhaps they should have even higher expectations. The payment of an Open Access fee has led to the proliferation of so-called predatory publishers with apparently exploitative business models. Some may charge fees to authors without providing effective peer review, for example, or the long-term archiving attendant to publication in valid academic journals. Over 8000 journals have now been identified as potentially predatory (https://scholarlyoa.files.wordpress.com/2015/01/criteria-2015.pdf).

The age of the predatory journal has in turn led to the launch of initiatives to help authors determine those Open Access journals that are sound and credible, and would provide a suitable home for their article. In 2008, the Open Access Scholarly Publishers Association (OASPA) was established. Its stated mission was to “represent the interests of Open Access (OA) journal publishers globally in all scientific, technical and scholarly disciplines”. To qualify for membership, BiO had to demonstrate adherence to OASPA membership criteria, and adopt its principles of transparency and best practice (http://oaspa.org/principles-of-transparency-and-best-practice-in-scholarly-publishing-2/). The Directory of Open Access Journals (DOAJ) is an online directory that lists high-quality, Open Access, peer-reviewed journals. The prevalence of predatory journals led the DOAJ to require all its listed journals to reapply for inclusion following assessment using more stringent criteria. BiO's reapplication was successful and the journal continues to be listed in the DOAJ. These respected associations are invaluable in helping authors safely identify those journals that are sound. BiO is a member of COPE (http://publicationethics.org/) and also supports the Think. Check. Submit. Campaign (http://thinkchecksubmit.org/), a simple checklist that researchers can use to assess the credentials of a journal or publisher.

## Journal and article metrics

Perhaps the factor that is most often considered by authors when choosing where to publish is the Impact Factor (IF). Despite much discussion of its limitations and flaws as a measure of the output of an individual researcher (Seglen, 1997; Kurmis, 2003; DePellegrin and Johnston, 2015), it remains a primary consideration when deciding where to submit an article. Start to Google the words ‘Biology Open’ and the second suggestion Google makes using its autocomplete search prediction is ‘Biology Open Impact Factor’, the autocomplete being based on what other people are searching for.

Well, we now have an IF and, although we are pleased with it, as signatories of the DORA agreement (http://www.ascb.org/dora/) we have undertaken to decrease emphasis on the journal IF as a promotional tool and instead present a range of journal- and article-level metrics to provide a richer view of journal performance (Raff, 2013). So, we have decided to showcase some of our ‘better-performing’ articles, according to different measures, but noting that these measures are perhaps all flawed in their own different ways.

Box 1 shows journal-level metrics for BiO and gives a brief explanation of their meaning. These citation-based metrics favour older articles, which of course have had more time to garner citations, and Review-type articles (which BiO does not publish). Table 1 shows the ten most-cited articles since journal launch.

**Box 1. Metrics and meanings.**

BiO uses a number of metrics that together provide a rich view of the journal's performance. These include:

• 2014 Impact Factor 2.416
• 5 Year Impact Factor 2.432
• Eigenfactor Score 0.00529
• Article Influence Score 1.124
• Cited half-life 2.0

**Impact Factor:** The average number of citations in a given year per paper published in the journal over the previous two years. Different subject areas exhibit different ranges of IF. Journals are best viewed in the context of their specific field.

**5 Year Impact Factor:** Offers a smoother variation and may be more appropriate in a field where the number of citations is small or where it takes longer than two years to disseminate and respond to published works.

**Eigenfactor Score:** A measure of the journal's influence within the network of academic citations with a five-year target window. Considers the origins of the incoming journal citations to articles in that journal. Citations from highly ranked journals are weighted to make a larger contribution to the Eigenfactor Score.

**Article Influence Score:** As for the Eigenfactor Score, measures influence but on an average per-article basis. The mean Article Influence Score is 1.00. A score greater than 1.00 indicates that the articles in a journal have an above average influence.

**Cited half-life:** A measure of how long content is referred to after publication. The number of years, going back from the current year, that account for half the total citations received by the journal in the current year.

**Table 1.**
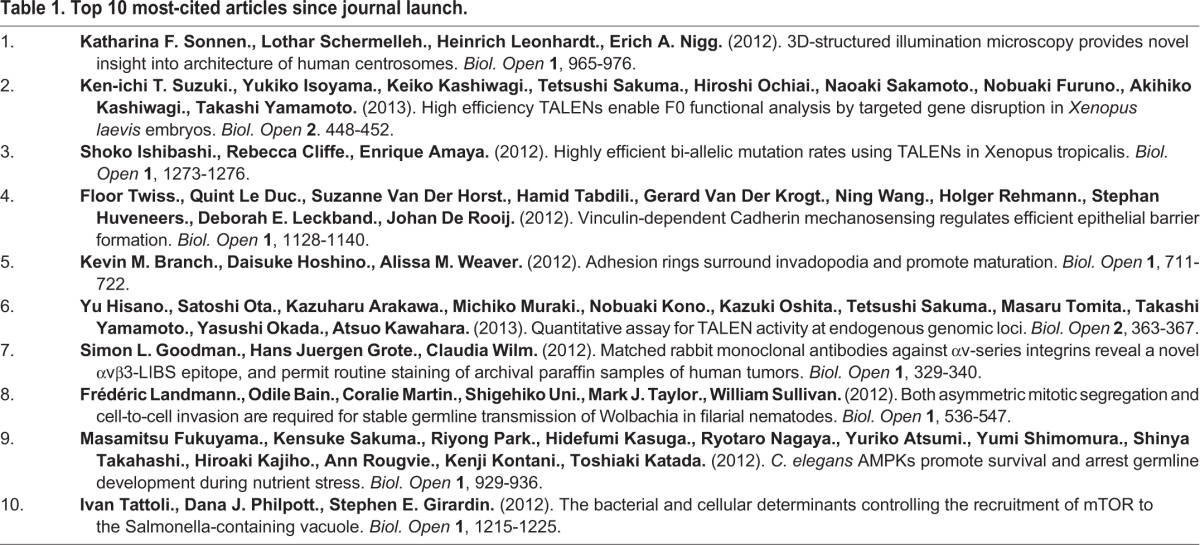
**Top 10 most-cited articles since journal launch.**

**Fig. 2.**
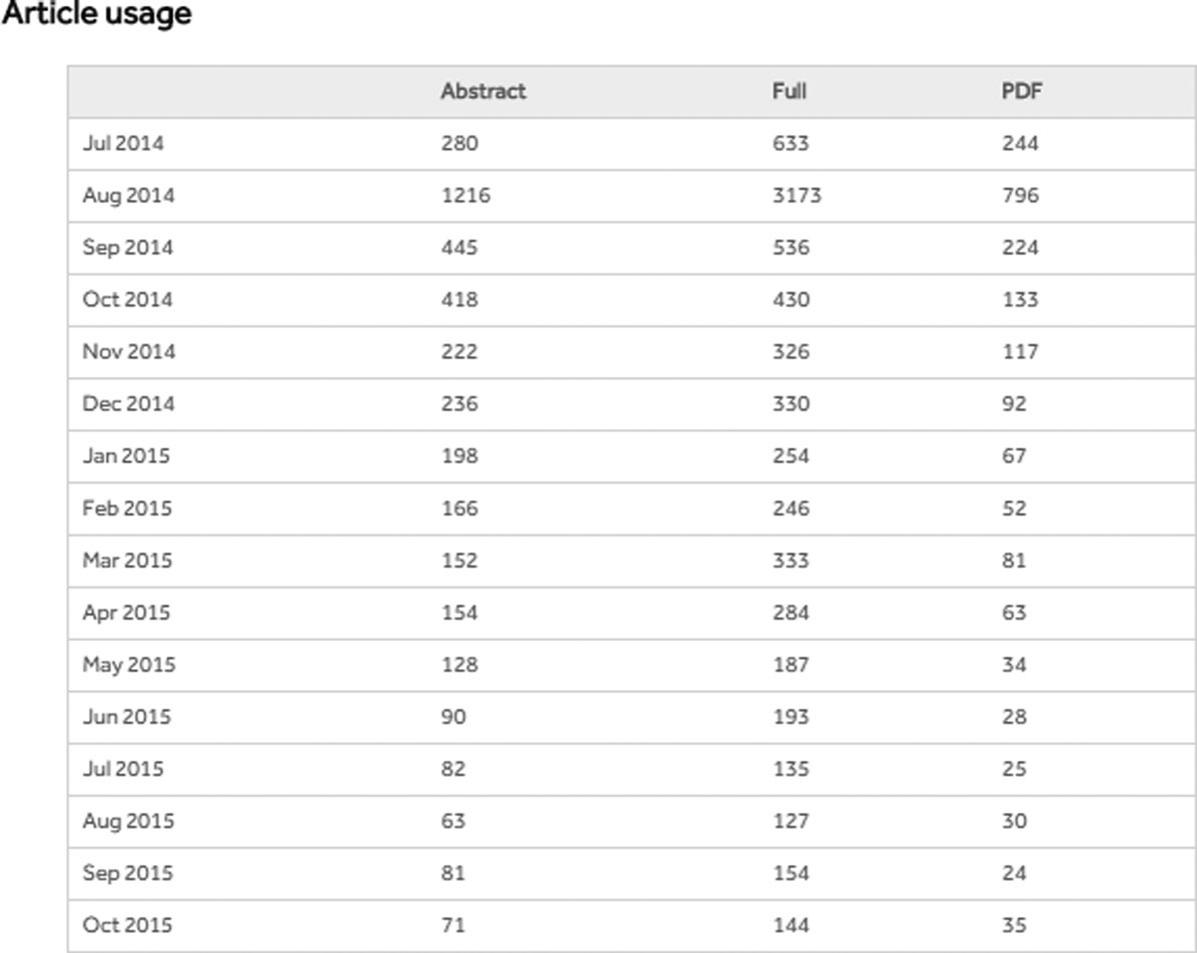
**Usage report for sample BiO article (Toyoda et al., 2014). Comparison of the usage reports for the different Company journals shows that BiO articles reach as many readers as those of our sister journals.**

BiO publishes Article Usage statistics, giving the online download statistics by month for abstract, full text and PDF views of a published article (Fig. 2). We also compile a monthly top 20 most-read article list based on full text and PDF views (http://bio.biologists.org/front.most-read). For BiO at least, only one of the top ten most read articles also features in the top ten most cited (see Table 2).

**Table 2.**
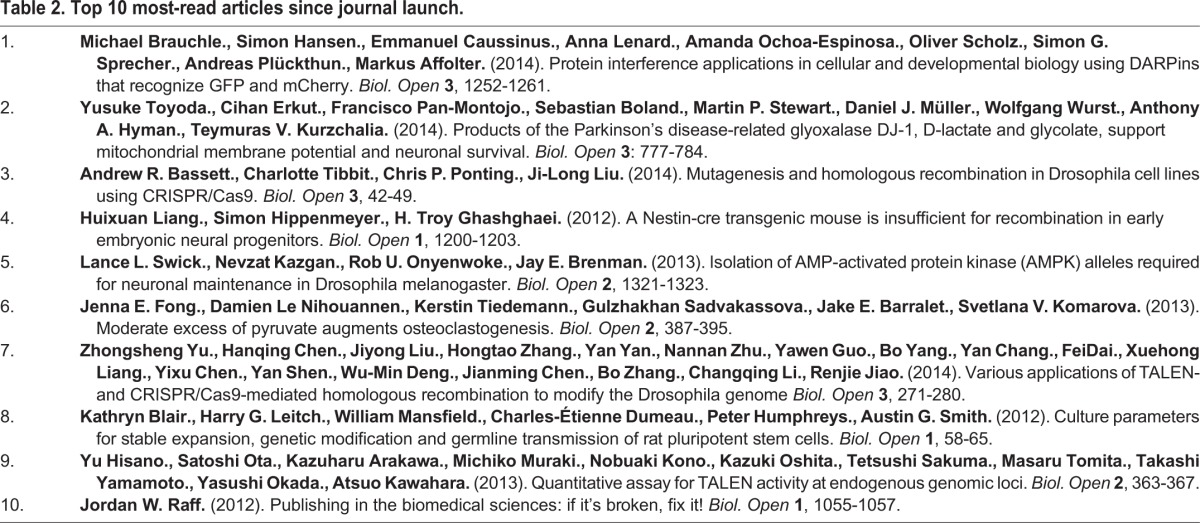
**Top 10 most-read articles since journal launch.**

The top ten most-read articles are a mixture of old and new articles. Those that are well read within the first two to six months after publication continue to be well read, and all-time reads are biased towards older articles (no big revelation). As expected for an Open Access journal, full text versions (HTML and PDF) attract high readership levels.

**Fig. 3.**
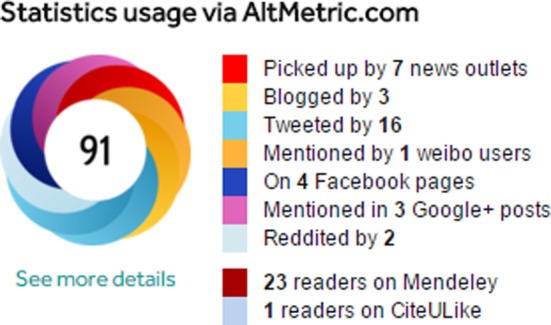
**The Altmetrics donut.**

**Fig. 4.**
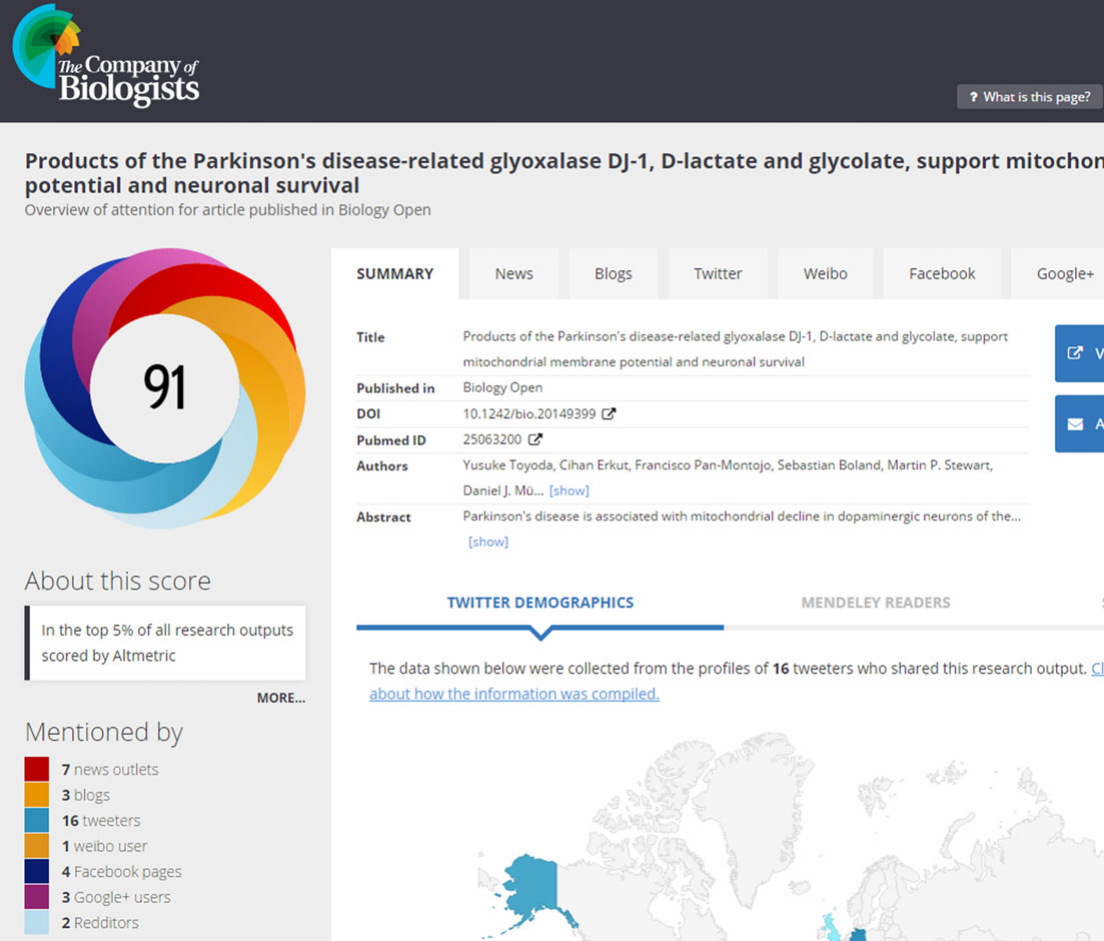
**Altmetric report for a sample BiO article (Toyoda et al., 2014), which is in the top 5% of all research outputs scored by Altmetric.**

Attention has turned recently to alternative article-level metrics or ‘Altmetrics’. Visitors to the BiO web site will see altmetric ‘donuts’ accompanying each article (Fig. 3). This colourful donut gives an at-a-glance summary of the online attention an article has received. The different colours of the donut represent the different sources in which the article has been mentioned (yellow, blogs; light blue, Twitter; red, news; dark blue, Facebook). Each article is given an Altmetric score; an overall measure of the quality and quantity of online attention that it has received (see Table 3). Categories are weighted to contribute a different amount to the overall score. For example, a news report contributes more than a blog mention, which contributes more than a Tweet. The source of the mention is also taken into account (see Fig. 4 for the Altmetric report for a sample BiO article; Toyoda et al., 2014).

**Table 3.**
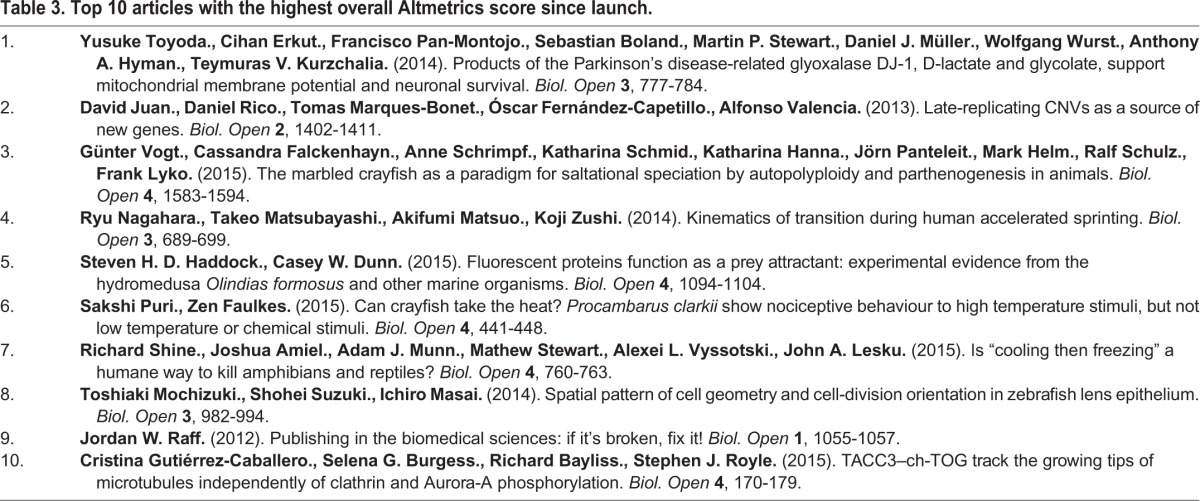
**Top 10 articles with the highest overall Altmetrics score since launch.**

Several articles that did not have a high Altmetrics score had high usage (HTML and PDF), although there is a correlation between those that have high scores in both datasets.

The top ten Altmetrics list contains a larger proportion of 2014 and 2015 (volumes 3 and 4) articles than other years for social media activity, including Reddit threads, blogs, Twitter, Google+ authors, Facebook walls and Weibo. Tweets are scored such that a tweet from BiO, say, counts for less than a tweet from someone unconnected with the paper. If a Nobel Prize winner finds the time to tweet about a paper, this would contribute more to the score. The top three papers had the most news outlet activity. The total number of ‘shares’ (via Mendeley and CiteULike) continues to accumulate, benefitting the older articles (Table 4). There is no overlap between the top ten most shared and the top ten highest overall Altmetrics score.

**Table 4.**
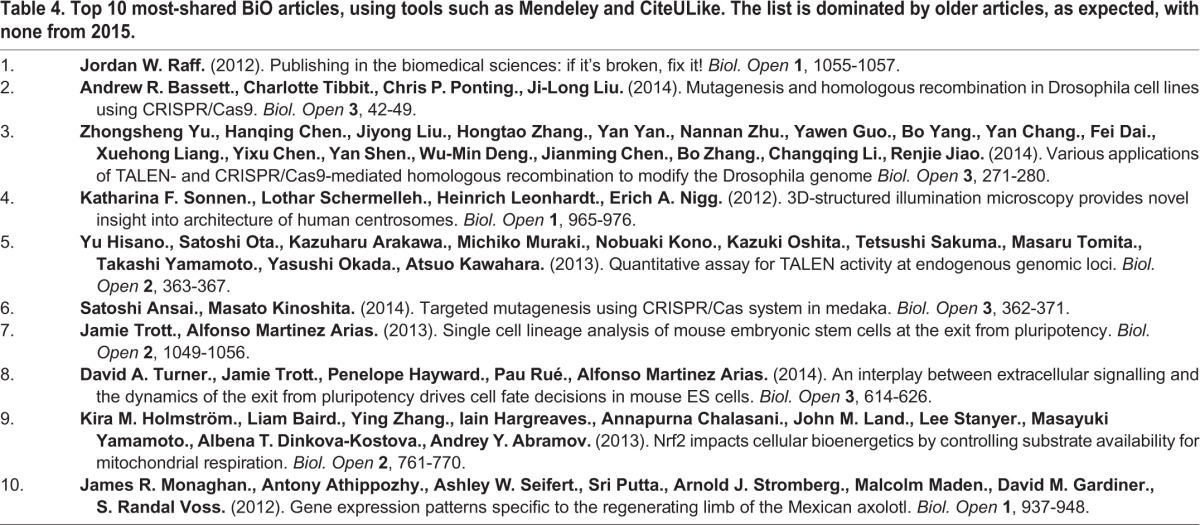
**Top 10 most-shared BiO articles, using tools such as Mendeley and CiteULike. The list is dominated by older articles, as expected, with none from 2015.**

A journal is more than its Impact Factor and a researcher is more than the journals in which they publish. Other articles have explained this more eloquently (http://scholarlykitchen.sspnet.org/2015/08/04/if-we-dont-know-what-citations-mean-what-does-it-mean-when-we-count-them/), but we hope that this article illustrates some of the contradictions and limitations of the different metrics in use today. These metrics together give an idea of some of the achievements of BiO to date; however, the statistic we are perhaps most proud of is that 100% of authors who responded to our author feedback survey would submit to BiO again. This and the testimonies we receive from authors confirm our belief that BiO is helping to make publishing good science both less painful and less time consuming for us all.
